# Prior treated tuberculosis and mortality risk in lung cancer

**DOI:** 10.3389/fmed.2023.1121257

**Published:** 2023-03-29

**Authors:** Kuang-Ming Liao, Chung-Shu Lee, Yu-Cih Wu, Chin-Chung Shu, Chung-Han Ho

**Affiliations:** ^1^Department of Internal Medicine, Chi Mei Medical Center, Chiali, Taiwan; ^2^Department of Pulmonary and Critical Care Medicine, New Taipei Municipal Tu Cheng Hospital, New Taipei City, Taiwan; ^3^Department of Thoracic Medicine, Chang Gung Memorial Hospital, School of Medicine, Chang Gung University, Taipei, Taiwan; ^4^Department of Medical Research, Chi Mei Medical Center, Tainan, Taiwan; ^5^Department of Internal Medicine, National Taiwan University Hospital and National Taiwan University College of Medicine, Taipei, Taiwan; ^6^Department of Information Management, Southern Taiwan University of Science and Technology, Tainan, Taiwan; ^7^Cancer Center, Taipei Municipal Wanfang Hospital, Taipei Medical University, Taipei, Taiwan

**Keywords:** lung cancer, mortality, tuberculosis, Taiwan Cancer Registry, risk factors

## Abstract

**Background:**

Lung cancer is one of the leading causes of cancer death worldwide, and tuberculosis (TB) is a common pre-existing disease. However, there is scarce literature studying the mortality risk in patients with prior TB and subsequent lung cancer.

**Methods:**

We recruited lung cancer patients from the Taiwan Cancer Registry from 2011 to 2015 and classified them into two groups according to presence or absence of prior TB. We then matched them in a ratio of 1:4 using the exact matching approach. The mortality risk within 3 years after diagnosis of lung cancer was analyzed and compared between these two groups.

**Results:**

During the study period, 43,472 patients with lung cancer were recruited, and of these, 1,211 (2.79%) patients had prior TB. After matching, this cohort included 5,935 patients with lung cancer in two groups: patients with prior TB before lung cancer (*n* = 1,187) and those without (*n* = 4,748). After controlling for demographic factors and comorbidities, the patients with prior TB had increased adjusted hazard ratios of 1.13 (95% CI: 1.04–1.23) and 1.11 (1.02–1.21) for all-cause and cancer-specific 3-year mortality, respectively, compared to the lung cancer patients without prior TB. Duration between TB and lung cancer (<1 year vs. 1–3 years vs. >3 years) had no differences for mortality risk.

**Conclusion:**

In the present study, 2.79% patients with lung cancer had prior TB, which was associated with higher 3-year mortality after they developed lung cancer. The mortality risk with prior TB did not decrease even if >3 years passed before diagnosis of lung cancer.

## Introduction

Lung cancer is the leading cause of cancer death in the world (1), and an estimated 1.8 million people died from lung cancer in 2020. Among the risk factors of lung cancer, a population cohort study showed that tuberculosis (TB) increased lung cancer risk (2, 3). In a Korean study, patients with previous pulmonary TB were also found to have a higher risk of lung cancer as compared with the general population (4). The speculation might be that increasing chronic inflammation in TB results in lung tissue damage, fibrosis, and scar formation (3). TB related dysregulation of inflammation cells and cytokines resulting from chronic pulmonary disorder can lead to unresolved cell damage and eliminate the ability of the tissue to repair itself (5, 6). The aforementioned mechanisms might thus predispose patients to lung cancer.

In addition to the association between TB and the risk of lung cancer, whether prior TB affects the lung cancer prognosis remains unclear. The possible speculations include chronic airway disease and decreased lung reserve complicated by TB (7, 8), as well as attenuated local immune status contributed by TB related inflammatory properties (9, 10). Although treated TB is reportedly associated with poor long-term survival as compared with the general population (11, 12), the influence of prior TB on short-term survival in subsequent lung cancer is unclear. Only one Korean study has reported a significant association of prior TB with lung cancer mortality (13), but no studies to date have replicated the results showing that prior TB leads to worse survival in patients with lung cancer. In addition, clinical stage and treatment modality were not included in the study’s adjustments. Therefore, we conducted this study to validate the influence of prior TB on 3-year mortality in patients with lung cancer by using detailed cancer data from the Taiwan Cancer Registry (TCR) and Taiwan’s National Health Insurance Research Database (NHIRD).

## Methods

### Data sources

The Taiwan Cancer Registry (TCR), which is linked with the National Health Insurance Research Database (NHIRD), was used to select patients with lung cancer and records of TB and comorbidities. For screening of the study subjects’ disease histories, administrative claims in the NHIRD from Taiwan’s National Health Insurance program, which cover all inpatient and outpatient health records, were also used in this study. Taiwan’s National Health Insurance program is a single-payer insurance program which covers almost 99% of the population of Taiwan (14). For research purposes, Taiwan’s Health and Welfare Data Science Center (HWDC) integrated the different health-related datasets and eliminated identifying data to avoid violations of personal information protection. This study was conducted in compliance with the Declaration of Helsinki and approved by the Ethics Committee of the Institutional Review Board of Chi-Mei Hospital (IRB: 10803-E01).

### Study population

Patients with new-onset lung cancer were enrolled in this study according to the International Classification of Diseases for Oncology, Third Edition (ICD-O-3) in TCR. The ICD-O-3 of lung cancer was C34. Since the TCR began recording behavioral information on smoking and drinking in 2011, patients from 2011 to 2015 were selected. After the exclusion of patients aged <20 years and those with missing data, the remaining 43,472 patients were selected.

In accordance with the aim of this study, patients with lung cancer were categorized as those with and without a history of prior TB (before diagnosis of lung cancer). The definition of TB was based on the ICD-9-CM codes, 010-012 for pulmonary TB and 013-018 for extrapulmonary TB, with at least a two separate diagnoses (within 6 months) for outpatients or one admission for inpatients. In addition, those without >6 weeks of first-line anti-TB drugs (at least two kinds) were excluded. First-line anti-TB drugs, including isoniazid (ATC codes: J04AC01, J04AC51, J04AM02, J04AM03, J04AM05, J04AM06, J04AM07), rifampin (ATC codes: J04AB02, J04AC51, J04AM02, J04AM05, J04AM06, J04AM07), pyrazinamide (ATC codes: J04AK01, J04AM05, J04AM06), and ethambutol (ATC codes: J04AK02, J04AM03, J04AM06, J04AM07), were also used to confirm the TB diagnosis to prevent misclassification bias. The drug code of second-line anti-TB drug were listed in the Supplementary File (Appendix 1).

### Matching

**Figure 1:**
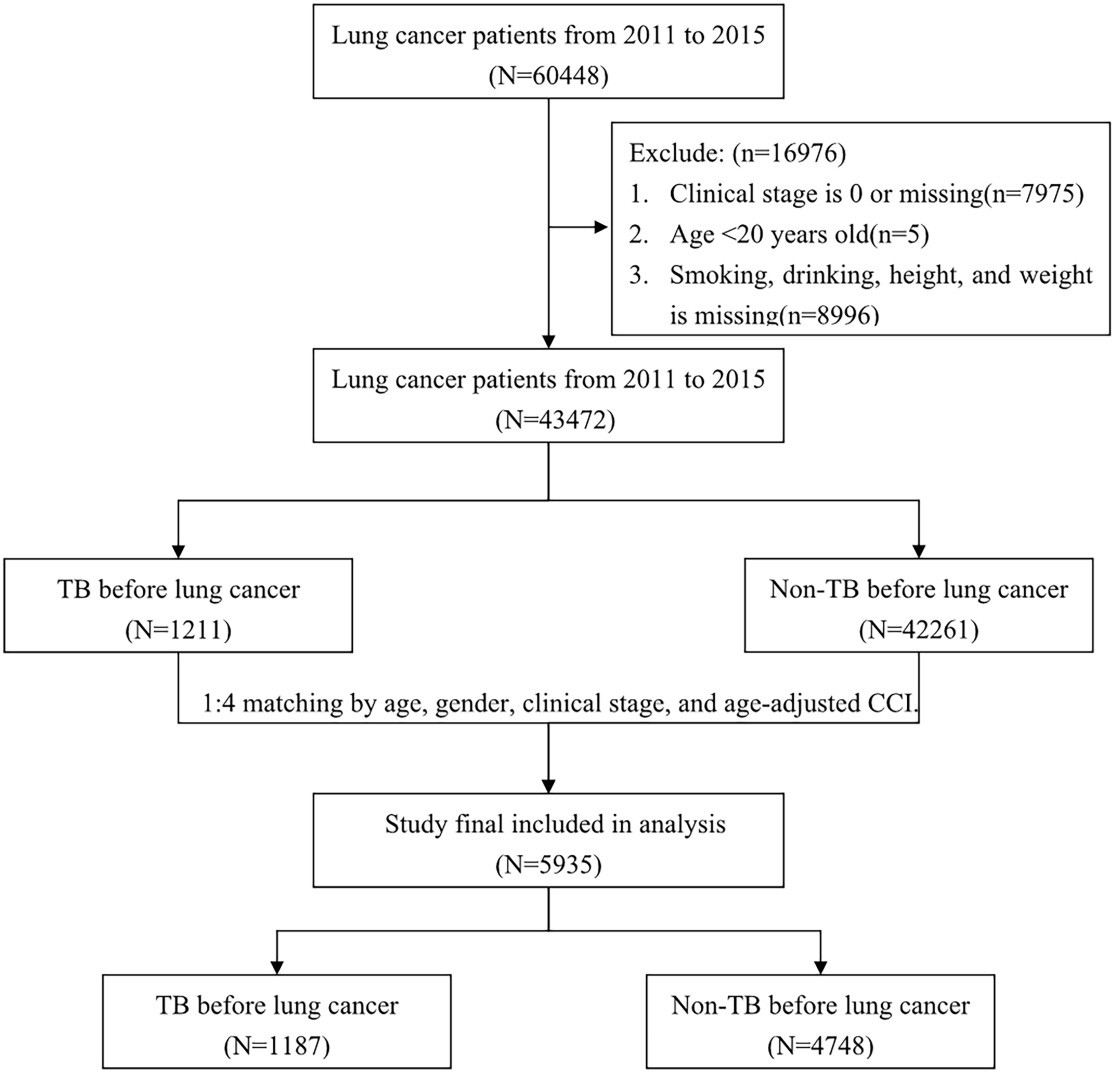
Flow chart of patient enrollment.

To decrease the confounding bias of mortality, we randomly matched each lung cancer patient with prior TB with four lung cancer patients without prior TB using the exact matching approach according to the variables of age, sex, clinical stage, and age-adjusted Charlson comorbidity index (CCI) score. After matching, a total of 5,935 patients with lung cancer (1,187 patients with TB history and 4,748 patients without TB history) were included in our study. A flowchart illustrating the selection of the study population is presented in Figure 1.

### Outcome and measurements

The major outcome of this study was overall mortality, which was identified as death due to any cause from Taiwan’s cause-of-death dataset. In addition, cancer-specific mortality was used to estimate the risk of mortality due to cancer. After the diagnosis date of lung cancer, all patients were followed up for 3 years, until death, or until the end date of the study, December 31, 2018.

For estimating the mortality risk, the potential confounding factors were considered as variables for adjustment, including types of cancer treatment received (operation, radiotherapy, chemotherapy), comorbidities, smoking, drinking, and body mass index (BMI). The comorbidities were identified using the ICD-9-CM and based on the 1-year medical records prior to the date of cancer diagnosis. Those comorbidities included diabetes mellitus (DM, ICD-9-CM:250), end-stage renal disease (ESRD, ICD-9-CM:585), chronic obstructive pulmonary disease (COPD, ICD-9-CM:490-492496), hypertension (ICD-9-CM: 401-405), ischemic heart disease (ICD-9-CM:410-414), and cerebrovascular disease (ICD-9-CM:430-438). Patients with smoking or drinking included both current and ever smokers/drinkers, respectively. BMI was categorized into three groups: underweight (<18.5 kg/m^2^), normal weight (18.5–25.0 kg/m^2^), and overweight (>25.00 kg/m^2^). CCI was established to evaluate the impact of comorbidities for mortality. Age-adjusted CCI (ACCI) scores were based on the CCI, but the ACCI scores considered the age effects in the assessment of the score; for patients aged >40 years, it increased by 1 for each decade (15, 16).

### Statistical analysis

The baseline distribution between patients diagnosed with TB and those without is presented as a frequency with percentages for categorical variables and mean with standard deviation for continuous variables. The difference in the above distribution was analyzed using Pearson’s chi-squared test for categorical variables and Student’s *t*-test for continuous variables. Kaplan–Meier curves were plotted to describe the proportions of patients who died due to all causes and due to cancer during the follow-up periods, and the log-rank test was used to compare risk differences between patients with TB and those without.

The overall and cancer-specific mortality risks were estimated in the matching cohorts using a Cox proportional regression model to calculate a crude hazard ratio (HR) with a 95% confidence interval (95% CI) The multivariable Cox regression model was used to estimate the adjusted HR (AHR) in the matching cohorts with adjustment for the potential confounding factors except those used for matching. The stratified analysis for overall and cancer-specific mortality in each comorbidity was also presented. Statistical significance was set to a two-tailed *p*-value of <0.05. All statistical analyses were performed in SAS (version 9.4; SAS Institute, Inc., Cary, NC, United States). Kaplan–Meier curves were plotted in STATA (version 12; Stata Corp., College Station, TX, United States).

## Results

### Patient enrollment and demographics

From 2011 to 2015, we reviewed 60,448 patients with diagnoses of lung cancer, and from that set, we finally analyzed 43,472 patients after excluding those with carcinoma *in situ* or missing data (*n* = 7,975), those aged <20 years (*n* = 5), and those without information on smoking, drinking, height, or weight (*n* = 8,996; Figure 1). In all, we identified 1,211 patients with prior TB before diagnosis of lung cancer and 42,261 patients without prior TB.

Patients with lung cancer and prior TB were predominantly male (77.37% vs. 58.88%) and older (70.02 (standard deviation [SD]: 12.05) years vs. 66.54 (12.47) years), and they were higher in ever smoking or drinking, lower in BMI, and higher (60.61% vs. 58.19%) in stage IV status (Table 1). The proportions which received chemotherapy and radiotherapy were comparable between the two groups, but operations were more common in those without prior TB. In addition, diabetes, COPD, and ESRD were more prevalent in those with prior TB, whereas hypertension, ischemic heart disease, and cerebrovascular disease were similarly distributed. Average age-adjusted CCI was higher in the prior TB group (7.23 vs. 6.03).

**Table 1:** Demographic characteristics and comorbidities of lung cancer patients without or with prior TB.

	Before match			After match*		
	Without TB (*N* = 42,261)	TB before lung cancer (*N* = 1,211)	Value of *p*	Without TB (*N* = 4,748)	TB before lung cancer (*N* = 1,187)	Value of *p*
Age, Mean ± SD	66.54 ± 12.47	70.02 ± 12.05	<0.0001	70.23 ± 11.66	70.24 ± 11.66	0.9987
Gender						
Male	24,885 (58.88)	937 (77.37)	<0.0001	3,688 (77.67)	922 (77.67)	1.0000
Female	17,376 (41.12)	274 (22.63)		1,060 (22.33)	265 (22.33)	
Smoking						
Never	22,448 (53.12)	451 (37.24)	<0.0001	1878 (39.55)	443 (37.32)	0.1586
Quit/Current	19,813 (46.88)	760 (62.76)		2,870 (60.45)	744 (62.68)	
Drinking						
Never	31,721 (75.06)	824 (68.04)	<0.0001	3,274 (68.96)	812 (68.41)	0.7156
Quit/Current	10,540 (24.94)	387 (31.96)		1,474 (31.04)	375 (31.59)	
BMI						
<18.5, kg/m^2^	3,755 (8.89)	187 (15.44)	<0.0001	469 (9.88)	185 (15.59)	<0.0001
18.5–25, kg/m^2^	25,641 (60.67)	785 (64.82)		2,883 (60.72)	765 (64.45)	
>25, kg/m^2^	12,865 (30.44)	239 (19.74)		1,396 (29.40)	237 (19.97)	
Clinical stage						
I	8,831 (20.90)	171 (14.12)	<0.0001	676 (14.24)	169 (14.24)	1.0000
II	1915 (4.53)	57 (4.71)		204 (4.30)	51 (4.30)	
III	6,922 (16.38)	249 (20.56)		952 (20.05)	238 (20.05)	
IV	24,593 (58.19)	734 (60.61)		2,916 (61.42)	729 (61.42)	
Treatment						
Operation, yes	12,360 (29.25)	256 (21.14)	<0.0001	1,032 (21.74)	254 (21.40)	0.8010
Radiotherapy, yes	12,302 (29.11)	359 (29.64)	0.6860	1,482 (31.21)	351 (29.57)	0.2732
Chemotherapy, yes	20,552 (48.63)	563 (46.49)	0.1417	2,412 (50.80)	553 (46.59)	0.0094
Comorbidity						
Diabetes	8,275 (19.58)	311 (25.68)	<0.0001	1,254 (26.41)	303 (25.53)	0.5355
COPD	6,964 (16.48)	459 (37.90)	<0.0001	1,175 (24.75)	448 (37.74)	<0.0001
ESRD	1,367 (3.23)	58 (4.79)	0.0027	260 (5.48)	56 (4.72)	0.2980
Hypertension	17,984 (42.55)	536 (44.26)	0.2364	2,372 (49.96)	527 (44.40)	0.0006
Ischemic Heart Disease	5,437 (12.87)	171 (14.12)	0.1988	797 (16.79)	170 (14.32)	0.0398
Cerebrovascular Disease	3,840 (9.09)	122 (10.07)	0.2389	652 (13.73)	119 (10.03)	0.0007
Age-adjusted CCI	6.03 ± 3.74	7.23 ± 3.67	<0.0001	7.18 ± 3.62	7.19 ± 3.62	0.9058
TB to cancer	–	2.26 ± 2.37		–	2.26 ± 2.38	
Time to follow up (within 3 year)	1.66 ± 1.16	1.27 ± 1.11	<0.0001	1.40 ± 1.13	1.27 ± 1.10	0.0004
3-year mortality	27,630 (65.38)	956 (78.94)	<0.0001	3,559 (74.96)	937 (78.94)	0.0042
3-year cancer-specific mortality	24,950 (59.04)	848 (70.02)	<0.0001	3,189 (67.17)	832 (70.09)	0.0536
Time to death (3 year)	0.95 ± 0.78	0.81 ± 0.74	<0.0001	0.87 ± 0.75	0.81 ± 0.73	0.0414

* Matching by gender, age (±1 year old), clinical stage, age-adjusted CCI (±1).

The mean duration of TB prior to diagnosis of lung cancer was 2.26 (SD: 2.37) years. The mean duration of follow-up was shorter (1.27 vs. 1.66 years) and 3-year mortality was higher (78.94% vs. 65.38% for all cause and 70.02 vs. 50.04 for cancer specific mortality) in patients with prior TB than in those without prior TB.

### Matched cohort

Due to many differences in demographics, we used the exact matching approach as a balancing score to reduce the effects of confounding factors. After 1:4 matching by gender, age, clinical stage and age-adjusted CCI, we finally enrolled 1,187 lung cancer patients with prior TB history and 4,748 lung cancer patients without TB history. There were no significant differences in gender, age, clinical stage, age-adjusted CCI group, smoking, or drinking between the matched cohorts. However, the prevalence of COPD was significantly higher and BMI was lower in the prior TB group than in the patients without TB. The prevalence of hypertension, ischemic heart disease and cerebrovascular disease were higher in the lung cancer without TB group than in the lung cancer with prior TB group.

### All-cause mortality in lung cancer patients with TB history in 3 years

**Figure 2:**
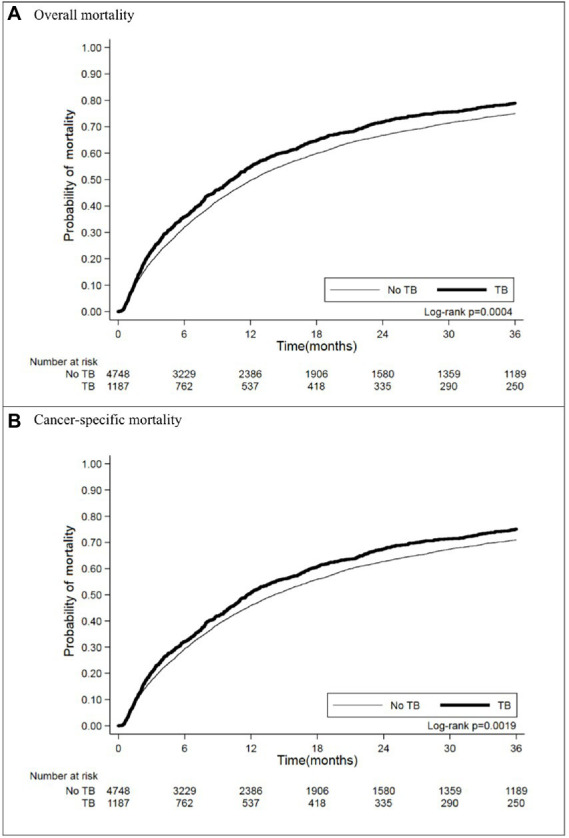
Kaplan–Meier curves of **(A)** overall and **(B)** cancer-specific mortality in matched cohort.

In matching the cohorts with an observation period of 3 years after diagnosis of lung cancer, 937 (78.94%) lung cancer patients with prior TB died, and their crude HR for all-cause mortality was 1.19 (95% CI [1.10–1.30]) (Table 2). The Kaplan–Meier curves (Figure 2A) showed that mortality was higher in the prior TB group than in the no prior TB group (*p* = 0.0004 by log rank test).

**Table 2:** The mortality risk of 3-year all-cause mortality among lung cancer patients.

	Patients	Death, No(%)	Crude HR	Value of *p*	AHR*	Value of *p*
Prior TB, yes (vs. no)	1,187	937 (78.94)	1.19 (1.10–1.30)	<0.0001	1.13 (1.04–1.23)	0.0044
Treatment						
Operation, yes (vs. no)	1,286	387 (30.09)	0.38 (0.33–0.45)	<0.0001	0.41 (0.35–0.47)	<0.0001
Radiotherapy, yes (vs. no)	1,833	1,543 (84.18)	1.12 (1.04–1.21)	0.0026	1.08 (1.00–1.17)	0.0614
Chemotherapy, yes(vs. no)	2,965	2,338 (78.85)	0.91 (0.84–0.98)	0.0109	0.88 (0.81–0.95)	0.0008
Comorbidity						
DM, yes (vs. no)	1,557	1,198 (76.94)	1.00 (0.92–1.10)	0.9459	1.10 (1.01–1.21)	0.0394
COPD, yes (vs. no)	1,623	1,329 (81.89)	1.13 (1.04–1.23)	0.0041	1.09 (0.99–1.19)	0.0756
ESRD, yes (vs. no)	316	260 (82.28)	1.09 (0.92–1.29)	0.3451	1.13 (0.95–1.34)	0.1715
HTN, yes (vs. no)	2,899	2,228 (76.85)	0.92 (0.85–0.99)	0.0211	0.99 (0.91–1.07)	0.7816
IHD, yes (vs. no)	967	763 (78.9)	0.97 (0.88–1.07)	0.5399	1.02 (0.92–1.13)	0.7250
CVD, yes (vs. no)	771	648 (84.05)	1.04 (0.94–1.16)	0.4637	1.05 (0.94–1.18)	0.3436
Smoking, quit/current (vs. never)	3,614	2,955 (81.77)	1.29 (1.18–1.41)	<0.0001	1.24 (1.13–1.37)	<0.0001
Drinking, quit/current (vs. never)	1,849	1,453 (78.58)	1.05 (0.97–1.13)	0.2206	0.98 (0.91–1.07)	0.6841
BMI						
<18.5 kg/m^2^	654	596 (91.13)	1.66 (1.49–1.86)	<0.0001	1.61 (1.43–1.80)	<0.0001
18.5–25 kg/m^2^	3,648	2,819 (77.28)	Ref.		Ref.	
>25 kg/m^2^	1,633	1,081 (66.2)	0.76 (0.70–0.82)	<0.0001	0.78 (0.71–0.85)	<0.0001

* Adjusted for treatment (operation, radiotherapy, and chemotherapy), comorbidities (DM, COPD, ESRD, HTN, IHD, and CVD), smoking, drinking, and BMI group.

After adjustments for factors not used in matching, including treatment (operation, radiotherapy, and chemotherapy), comorbidities (including DM, COPD, ESRD, HTN, IHD, and CVD), smoking, drinking, and BMI, prior TB was associated with an AHR of 1.13 (95% CI [1.04–1.23]). Patients who received operations had a crude HR of 0.38 (95% CI [0.33–0.45]) and an AHR of 0.41 (95% CI [0.35–0.47]), and those with chemotherapy had a crude HR of 0.91 (95% CI [0.84–0.98]) and an AHR of 0.88 (95% CI [0.81–0.95]). The patients with diabetes mellitus had higher mortality, with an AHR of 1.10 (95% CI [1.01–1.21]), and smoking was associated with an AHR of 1.24 (95% CI [1.13–1.37]). Low BMI (< 18.5 kg/m^2^) was associated with increased mortality (AHR: 1.61, 95% CI: 1.43–1.80), but high BMI (>25 kg/m^2^) was associated with decreased AHR (0.78, 95% CI: 0.71–0.75).

### Cancer-specific mortality in lung cancer patients

We further analyzed the lung cancer-specific mortality and found that prior TB increased lung cancer mortality, as indicated by the increased crude HR (1.18 [1.08–1.28]) (Table 3) and worse cumulative mortality curve (Figure 2B, *p* = 0.0019 by log rank test). Multivariable Cox proportional regression showed that prior TB was an independent factor for 3-year cancer-specific mortality (AHR: 1.11 [1.02–1.21]) (Table 3). The different treatment modalities were significantly associated with lung cancer-specific mortality risk (operation [AHR: 0.37, 95% CI: 0.32–0.44]; radiotherapy [AHR: 1.12, 95% CI: 1.03–1.21]; and chemotherapy [AHR: 0.90, 95% CI: 0.83–0.98]). The patients with underlying comorbidities of DM and COPD had higher mortality rates, with an AHR of 1.15 (95% CI [1.04–1.27]) and 1.12 (95% CI [1.02–1.24]), respectively. The effect of smoking on cancer-specific mortality was significant (AHR: 1.23, 95% CI [1.11–1.36]). BMI < 18.5 kg/m^2^ was associated with increased lung cancer mortality risk (AHR: 1.58, 95% CI [1.40–1.78]), and BMI > 25 kg/m^2^, with decreased mortality risk (AHR: 0.77, 95% CI [0.70–0.84]) compared with BMI of 18.5–25 kg/m^2^.

**Table 3:** The mortality risk of 3-year cancer-specific mortality among lung cancer patients.

	Patients	Death, No (%)	Crude HR	Value of *p*	AHR*	Value of *p*
Prior TB, yes (vs. no)	1,187	832 (70.09)	1.18 (1.08–1.28)	0.0002	1.11 (1.02–1.21)	0.0204
Treatment						
Operation, yes (vs. no)	1,286	297 (23.09)	0.35 (0.29–0.41)	<0.0001	0.37 (0.32–0.44)	<0.0001
Radiotherapy, yes (vs. no)	1,833	1,418 (77.36)	1.16 (1.08–1.26)	0.0002	1.12 (1.03–1.21)	0.0084
Chemotherapy, yes (vs. no)	2,965	2,131 (71.87)	0.94 (0.87–1.02)	0.1119	0.90 (0.83–0.98)	0.0131
Comorbidity						
DM, yes (vs. no)	1,557	1,070 (68.72)	1.04 (0.94–1.14)	0.4679	1.15 (1.04–1.27)	0.0059
COPD, yes (vs. no)	1,623	1,193 (73.51)	1.17 (1.07–1.28)	0.0008	1.12 (1.02–1.24)	0.0176
ESRD, yes (vs. no)	316	217 (68.67)	1.04 (0.86–1.25)	0.7183	1.08 (0.90–1.31)	0.4054
HTN, yes (vs. no)	2,899	1955 (67.44)	0.91 (0.84–0.98)	0.0128	0.97 (0.90–1.06)	0.5209
IHD, yes (vs. no)	967	669 (69.18)	0.96 (0.87–1.07)	0.4931	1.02 (0.92–1.14)	0.7031
CVD, yes (vs. no)	771	585 (75.88)	1.06 (0.95–1.19)	0.2934	1.09 (0.97–1.22)	0.1646
Smoking, quit/current (vs. never)	3,614	2,632 (72.83)	1.29 (1.17–1.41)	<0.0001	1.23 (1.11–1.36)	<0.0001
Drinking, quit/current (vs. never)	1,849	1,291 (69.82)	1.04 (0.96–1.13)	0.3816	0.97 (0.89–1.06)	0.5147
BMI						
<18.5 kg/m^2^	654	525 (80.28)	1.63 (1.45–1.84)	<0.0001	1.58 (1.40–1.78)	<0.0001
18.5–25 kg/m^2^	3,648	2,537 (69.54)	Ref.		Ref.	
>25 kg/m^2^	1,633	959 (58.73)	0.75 (0.68–0.82)	<0.0001	0.77 (0.70–0.84)	<0.0001

* Adjusted for treatment (operation, radiotherapy, and chemotherapy), comorbidities (DM, COPD, ESRD, HTN, IHD, and CVD), smoking, drinking, and BMI group.

### The role of treatment of prior TB, cancer type, and TB type on the survival of lung cancer

Regarding different treatment for prior TB, whether they had received second line anti-TB regimen did not influence the all-cause mortality (AHR: 1.22, 95% CI [0.96–1.30]) but increased cancer specific morality (AHR: 1.18 [1.00–1.38]) compared with those received all first-line anti-TB regimen (Supplementary Table S1 in the Supplementary File).

For different type of lung cancer pathology, small cell lung cancer (*n* = 586) had higher all-cause mortality (AHR: 1.49 [1.33–1.67]) and cancer-specific mortality (AHR: 1.47 [1.31–1.66]) than those with non-small cell lung cancer (*n* = 5,349; Supplementary Table S2 in the Supplementary File). In addition, we classified the types of prior TB as pulmonary and extra-pulmonary TB. The lung cancer patients with prior pulmonary TB presented significant worse all-cause (AHR: 1.10, 95% CI [1.02–1.18]) and cancer-specific mortality (AHR: 1.09, 95% CI [1.00–1.18]) compared with those without TB (Supplementary Table S2). However, the all-cause and cancer specific mortality was not significantly different between lung cancer patients with extra-pulmonary and those without TB (AHR: 1.00 [0.73–1.36] and 0.94 [0.67–1.33], respectively). The influence of pulmonary TB on the survival of lung cancer patients were still significant higher in the patients with non-small cell lung cancer but not significant in those with small cell lung cancer.

### The role of duration between prior TB to lung cancer

Among patients with prior TB and lung cancer, we further divided the durations between prior TB to lung cancer into three groups, namely, <1 year (*n* = 541, 46%), 1–3 years (*n* = 237, 20%), and >3 years (*n* = 409, 34%), to investigate the effect of the length of time from TB to lung cancer on the later mortality risk (Table 4). We found that, compared with prior TB > 3 years before lung cancer, TB to lung cancer of 1–3 years or <1 year was not associated with a significant difference in mortality risk (Table 4). The duration of prior TB had no significant influence on mortality. There were also no significant differences in gender, age, or cancer stage for the mortality risk by the duration from prior TB to lung cancer.

**Table 4:** The risk of 3-year all cause and cancer-specific mortality among lung cancer patients with TB stratified by different clinical factors.

	All-cause mortality				Cancer-specific mortality		
	Patients	Death, No (%)	AHR*	Value of *p*	Death, No (%)	AHR*	Value of *p*
Duration, TB to lung cancer							
<1 year	541	418 (77.26)	1.02 (0.87–1.18)	0.8469	384 (70.98)	1.08 (0.92–1.26)	0.3726
1–3 years	237	187 (78.9)	0.96 (0.80–1.15)	0.6435	160 (67.51)	0.95 (0.78–1.15)	0.5808
>3 years	409	332 (81.17)	Ref.		288 (70.42)	Ref.	
Stratified							
Male							
<1 year	394	327 (82.99)	1.00 (0.84–1.18)	0.9326	301 (76.4)	1.07 (0.90–1.28)	0.4262
1–3 years	195	160 (82.05)	0.88 (0.72–1.07)	0.1964	137 (70.26)	0.87 (0.71–1.08)	0.2149
>3 years	333	281 (84.38)	Ref.		241 (72.37)	Ref.	
Female							
<1 year	147	91 (61.9)	1.24 (0.83–1.85)	0.2933	83 (56.46)	1.20 (0.79–1.83)	0.4001
1–3 years	42	27 (64.29)	1.66 (0.99–2.77)	0.0539	23 (54.76)	1.51 (0.87–2.61)	0.1418
>3 years	76	51 (67.11)	Ref.		47 (61.84)	Ref.	
Age < 50							
<1 year	53	34 (64.15)	0.68 (0.22–2.08)	0.5012	34 (64.15)	0.67 (0.22–2.03)	0.4777
1–3 years	10	6 (60)	0.58 (0.13–2.63)	0.4827	5 (50)	0.51 (0.11–2.36)	0.3891
>3 years	9	6 (66.67)	Ref.		6 (66.67)	Ref.	
50–59							
<1 year	88	61 (69.32)	0.78 (0.45–1.36)	0.3842	57 (64.77)	0.92 (0.51–1.66)	0.7925
1–3 years	19	15 (78.95)	1.22 (0.60–2.45)	0.5837	14 (73.68)	1.24 (0.59–2.60)	0.5677
>3 years	38	26 (68.42)	Ref.		23 (60.53)	Ref.	
60–69							
<1 year	137	97 (70.8)	1.09 (0.78–1.52)	0.6088	91 (66.42)	1.17 (0.82–1.67)	0.3795
1–3 years	59	39 (66.1)	0.83 (0.55–1.26)	0.3821	33 (55.93)	0.80 (0.51–1.25)	0.3280
>3 years	95	67 (70.53)	Ref.		59 (62.11)	Ref.	
Age ≥ 70							
<1 year	263	226 (85.93)	1.07 (0.89–1.29)	0.4970	202 (76.81)	1.12 (0.92–1.37)	0.2572
1–3 years	149	127 (85.23)	1.00 (0.80–1.25)	0.9830	108 (72.48)	0.99 (0.78–1.26)	0.9370
>3 years	267	233 (87.27)	Ref.		200 (74.91)	Ref.	
Early stage							
<1 year	92	30 (32.61)	1.07 (0.64–1.80)	0.8039	27 (29.35)	1.46 (0.82–2.59)	0.1987
1–3 years	49	16 (32.65)	0.62 (0.34–1.14)	0.1230	12 (24.49)	0.60 (0.29–1.21)	0.1507
>3 years	79	37 (46.84)	Ref.		27 (34.18)	Ref.	
Last stage							
<1 year	449	388 (86.41)	1.01 (0.86–1.18)	0.9046	357 (79.51)	1.05 (0.89–1.24)	0.5786
1–3 years	188	171 (90.96)	1.03 (0.85–1.25)	0.7562	148 (78.72)	1.01 (0.83–1.24)	0.8956
>3 years	330	295 (89.39)	Ref.		261 (79.09)	Ref.	

* Adjusted for gender, age group, clinical stage, age-adjusted CCI group, treatment (operation, radiotherapy, and chemotherapy), comorbidities (DM, COPD, ESRD, HTN, IHD, and CVD), smoking, drinking, and BMI group.

## Discussion

In the present study, prior TB was found in 2.79% patients with lung cancer and was associated with increased mortality risk (AHR: 1.13 for all-cause death and 1.11 for cancer-specific mortality) in patients with lung cancer. The duration from prior TB to lung cancer was highest for <1 year (46%) and was not correlated with a difference in mortality risk. Other independent factors for mortality risk in lung cancer beyond those used for matching included treatment modality, smoking, DM, COPD for cancer specific mortality only, and BMI.

TB and lung cancer were highly correlated with each other. Consistent with a previous report of a high TB incidence in lung cancer (17), our study found a rate of 2.79% in lung cancer patients. A systematic review and meta-analysis also showed that preexisting pulmonary TB was significantly associated with lung cancer, with OR 2.09 (95% CI: 1.62–2.69) (18). Moreover, TB might induce a decline in lung function (8) and inflammation (19) and may lead to chronic airway disease (7). Therefore, the mortality of patients treated for TB is higher than that of the general population in a life-long estimation (11, 12). However, this issue has been scarcely discussed with a focus on patients treated for TB and subsequently for lung cancer. Only a retrospective cohort study that enrolled Korean adults in 1997–2000 showed that TB was significantly associated with lung cancer mortality (HR 1.43 in men with 95% CI 1.34–1.52; HR 1.53 in women with 95% CI 1.28–1.83) (13). Before the present study, no other studies have replicated this finding on TB related mortality risk in lung cancer. In addition to smoking, the present study analyzed BMI and the underlying comorbidities of diabetes mellitus, COPD, ESRD, HTN, IHD, CVD and CCI because they are vital confounding factors for mortality. Moreover, we included cancer stage and treatment modality from the TCR database, as they also strongly affect cancer mortality. We used age, gender, clinical stage and ACCI for matching and analyzed the impact of prior TB on lung cancer mortality by Cox proportional regression using adjustments for treatment modality, comorbidities, smoking, and BMI. The results showed that prior TB significantly increased the 3-year mortality rate in patients with subsequent lung cancer, which echoed the previous Korean study (13), but the impact found in this study was smaller, possibly due to the detailed correction for variables.

In the subgroup analysis, the negative effect or prior TB on the survival of subsequent lung cancer might majorly come from those received second-line anti-TB regimen, with pulmonary type of TB, or with pathology type of non-small cell lung cancer. Those received second-line anti-TB regimen might indicate their treatment complexity or TB resistance, which are associated with more underlying disease or poor prognosis (20). Because the case number of prior extra-pulmonary TB was small, the insignificant impact of extra-pulmonary TB on the lung cancer survival might need further large-scale study to validate. On the other hand, prior pulmonary TB remained independent factor associated with worse survival and confirmed our study result. For cancer subtype, small cell lung cancer, which was associated with higher mortality than non-small cell lung cancer, was similar as previous report (21). Under such high mortality in small cell lung cancer, the impact of prior TB might be masked and not significant. By contrast, the effect of prior pulmonary TB on the survival was persistently significantly in patients with non-small cell lung cancer.

We divided duration from prior TB to diagnosis of lung cancer into three groups: <1 year, 1–3 years and >3 years. Although the <1-year subgroup accounted for the largest proportion (46%), there was no significant difference in mortality risk among the three subgroups. This finding might indicate that the effect of prior TB was not only significant for increased mortality in subsequent lung cancer but also persistently affected the mortality risk for more than 3 years. Future bundle care should incorporate prior TB as a risk for lung cancer screening and comprehensive care in patients with subsequent lung cancer.

Some possible underlying mechanisms may explain this effect of prior TB on mortality in lung cancer. Firstly, TB is an infectious disease which most often occurs in patients with impaired immune systems (22, 23). To fight against tumor cells, T cells should increase their aggregation and migration efficiency to defend against tumor cells. Unfortunately, there is increasing evidence that T cells present dysfunction and migrate into tumor cells, which prevents the former from killing the latter (24). Therefore, lung cancer patients with prior TB may have attenuation of efficient T cells to kill tumor cells and thus poor prognosis. Second, TB is a risk factor of COPD through several mechanisms. Patients with TB present recurrent inflammation and residual inflammation, which can lead to lung destruction and emphysematous change. During the inflammation process, macrophage dysfunction also plays an important role in airway remodeling, leading to chronic airflow obstruction (25, 26). In addition, pulmonary fibrotic change, scarring, and lung parenchymal destruction due to TB can also cause airway obstruction (27). Patients with concurrent obstructive lung disease and lung cancer were at higher risk of reduced survival (28) and postoperative complications (29).

Other factors that influenced lung cancer mortality were similar to those in previous reports. The treatment modality for lung cancer might be indirectly correlated with its survival. For example, operation indicates the early stage, while radiotherapy usually represents the late stage (30). Smoking, low BMI, and underlying diseases of DM and COPD were adversely correlated with lung cancer outcome (31–33). Our study similarly found that smoking history, presence of COPD and DM, and BMI < 18.5 were positively associated with mortality, but high BMI had decreased mortality in patients with lung cancer.

There are several limitations in our study. First, this was a retrospective study design using claimed database, and some detailed information, such as pulmonary function and laboratory data as well as molecular data of lung cancer, were unavailable. In addition, individual treatment was not standardized, and data on self-paid regimens were not included in the database. Third, after we had performed the cohort matching, there were several underlying diseases significantly different between the two groups. Although we performed the multivariable regression to adjust these factors, they might still lead a bias. Last, the study was conducted in Taiwan, a TB prevalent area. The results should be validated in different areas and ethnicities before further generalization.

In conclusion, in patients with lung cancer, the rate of prior TB was 2.79%, and 46% of the cases occurred within the year preceding the diagnosis of lung cancer. Prior TB was associated with increased 3-year mortality (all-cause or cancer-specific) after lung cancer in multivariable Cox proportional regression. Duration between diagnosis of TB and lung cancer was not correlated with mortality, indicating that prior TB might affect the mortality of lung cancer patients even >3 years after treatment.

## Data availability statement

The data analyzed in this study is subject to the following licenses/restrictions: Data are available from the Taiwan Cancer Registry. Due to legal restrictions imposed by the government of Taiwan in relation to the “Personal Information Protection Act,” data cannot be made publicly available. Requests to access these datasets should be directed to https://twcr.tw/?page_id=1843&lang=en.

## Author contributions

C-HH and Y-CW collected and analyzed data. K-ML, C-SL, C-CS, and C-HH performed data analysis and wrote the manuscript, as well as providing critical revisions to the paper. C-CS and C-HH were the guarantors of the paper and taking responsibility for the integrity of the work. All authors contributed to the article and approved the submitted version.

## Funding

This study was partially funded by grants from National Taiwan University (NTUH 112-S0071) and the Ministry of Science and Technology, Taiwan (MOST 109-2326-B-002-009-MY3).

## Conflict of interest

The authors declare that the research was conducted in the absence of any commercial or financial relationships that could be construed as a potential conflict of interest.

## Publisher’s note

All claims expressed in this article are solely those of the authors and do not necessarily represent those of their affiliated organizations, or those of the publisher, the editors and the reviewers. Any product that may be evaluated in this article, or claim that may be made by its manufacturer, is not guaranteed or endorsed by the publisher.
