# Supplementary material for: Prior treated tuberculosis and mortality risk in lung cancer
Source: Front Med (Lausanne). 2023 Mar 29;10:1121257. doi: 10.3389/fmed.2023.1121257 (PMC10090669; doi:10.3389/fmed.2023.1121257)
Supplement: Supplementary file 1 [file Data_Sheet_1.DOCX]

**Appendix 1.** The drug ATC-codes of first-line or second-line anti-TB.

First-line drugs:

- Ethambutol (J04AK02, J04AM03, J04AM07, J04AM06)
- Isoniazid (J04AC01, J04AM03, J04AC51, J04AM02, J04AM07, J04AM05, J04AM06)
- Pyrazinamide (J04AK01, J04AM05, J04AM06)
- Rifampicin (J04AB02, J04AM02, J04AM07, J04AM05, J04AM06)

Second-line drugs:

- Amikacin (J01GB06), Clofazimine(J04BA01), Cycloserine (J04AB01), Kanamycin(J01GB04), Ciprofloxacin(J01MA02), Levofloxacin(J01MA12), Moxifloxacin(J01MA14), Protionamide (J04AD01).

**Table S1.** The risk of all-cause and cancer-specific mortality among lung cancer patients with prior TB according to the different treatment for prior TB.

|  | All-cause mortality | | Cancer-specific mortality | |
| --- | --- | --- | --- | --- |
|  | Death | AHR(95%CI) | Death in LC | AHR(95%CI) |
| Type of anti-TB drug | |  |  |  |
| All first-line | 711(77.37) | Ref. | 625(68.01) | Ref. |
| Including Second-line | 226(84.33) | 1.12(0.96-1.30) | 207(77.24) | 1.18 (1.00-1.38) ^*^ |

Adjusted for age, gender, clinical stage, CCI group, treatment(operation, radiotherapy, and chemotherapy), comorbidities(DM, COPD, ESRD, HTN, IHD, and CVD), smoking, drinking, and BMI group.

^*^ P<0.05

**Table S2**. The risk of three-year all-cause and cancer-specific mortality among lung cancer patients with different subtype of lung cancer or prior tuberculosis (TB).

|  | **All-cause mortality** | | **Cancer specific mortality** | |
| --- | --- | --- | --- | --- |
|  | Death  (N=4496) | AHR^#^(95%CI) | Death in LC  (N=4021) | AHR^#^ (95% CI) |
| **All study subjects** |  |  |  |  |
| Lung cancer subtype |  |  |  |  |
| NSCLC, n=5349 | 3947(73.79) | Ref. | 3521(65.83) | Ref. |
| SCLC, n=586 | 549(93.69) | 1.49(1.33-1.67)* | 500(85.32) | 1.47(1.31-1.66)* |
| TB subtype |  |  |  |  |
| pulmonary TB, n=1126 | 897(79.66) | 1.10(1.02-1.18)* | 799(70.96) | 1.09(1.00-1.18)* |
| extrapulmonary TB, n=61 | 40(65.57) | 1.00(0.73-1.36) | 33(54.10) | 0.94(0.67-1.33) |
| Without TB, n=4748 | 3559(74.96) | Ref. | 3189(67.17) | Ref. |
| **Patients with SCLC** |  |  |  |  |
| TB subtype |  |  |  |  |
| without TB, n=4748 | 456(93.63) | Ref. | 420(86.24) | Ref. |
| pulmonary TB, n=92 | 87(94.57) | 1.06(0.83-1.34) | 77(83.70) | 1.00(0.78-1.28) |
| extrapulmonary TB, n=7 | 6(85.71) | 0.70(0.31-1.61) | 3(42.86) | 0.38(0.12-1.22) |
| **Patients with NSCLC** |  |  |  |  |
| TB subtype |  |  |  |  |
| Without TB, n=4748 | 3103(72.82) | Ref. | 2769(64.98) | Ref. |
| pulmonary TB, n=1034 | 810(78.34) | 1.12(1.04-1.22)* | 722(69.83) | 1.12(1.03-1.21)* |
| extrapulmonary TB, n=54 | 34(62.96) | 0.97(0.69-1.37) | 30(55.56) | 0.98(0.69-1.41) |

^#^Adjusted for age, gender, clinical stage, CCI group, treatment (operation, radiotherapy, and chemotherapy), comorbidities (DM, COPD, ESRD, HTN, IHD, and CVD), smoking, drinking, and BMI group.

^*^ P<0.05

Abbreviations: NSCLC, non-small cell lung cancer; SCLC, small cell lung cancer; TB, tuberculosis.
